# EULAR points to consider for including the perspective of young patients with inflammatory arthritis into patient-reported outcomes measures

**DOI:** 10.1136/rmdopen-2022-002576

**Published:** 2022-07-29

**Authors:** Paul Studenic, Tanja A Stamm, Erika Mosor, Ilaria Bini, Nele Caeyers, Laure Gossec, Marios Kouloumas, Elena Nikiphorou, Wendy Olsder, Ivan Padjen, Sofia Ramiro, Simon Stones, Tanita-Christina Wilhelmer, Alessia Alunno

**Affiliations:** 1 Department of Internal Medicine 3, Division of Rheumatology, Medical University of Vienna, Wien, Austria; 2 Department of Medicine (Solna), Division of Rheumatology, Karolinska Institute, Stockholm, Sweden; 3 Ludwig Boltzmann Institut für Arthritis und Rehabilitation, Wien, Austria; 4 Section for Outcomes Research, Center for Medical Statistics, Informatics and Intelligent Systems, Medical University of Vienna, Vienna, Austria; 5 Anmar Young, Rome, Italy; 6 EULAR Young PARE, Zürich, Switzerland; 7 EULAR PARE, Zürich, Switzerland; 8 INSERM, Institut Pierre Louis d'Epidémiologie et de Santé Publique, INSERM, Sorbonne Universite, Paris, France; 9 AP-HP.Sorbonne Université, Rheumatology department, Hopital Universitaire Pitie Salpetriere, Paris, France; 10 Cyprus League Against Rheumatism, Aglantzia, Cyprus; 11 Centre for Rheumatic Diseases, King's College London, London, UK; 12 Rheumatology Department, King's College Hospital, London, UK; 13 Youth-R-Well, Nieuwegein, The Netherlands; 14 Department of Internal Medicine, Division of Clinical Immunology and Rheumatology, University Hospital Centre Zagreb, Zagreb, Croatia; 15 Department of Rheumatology, Leiden University Medical Center, Leiden, The Netherlands; 16 Rheumatology, Zuyderland Medical Centre Heerlen, Heerlen, The Netherlands; 17 Envision Pharma Group Limited, Wilmslow, UK; 18 Österreichische Rheumaliga, Maria Alm, Austria; 19 Department of Life, Health and Environmental Sciences, University of L’Aquila, L'Aguila, Italy; 20 Internal Medicine and Nephrology Unit, Department of Medicine, ASL Avezzano-Sulmona-L'Aquila, San Salvatore Hospital, L'Aquila, Italy

**Keywords:** arthritis, patient reported outcome measures, outcome assessment, health care

A range of patient-reported outcome measures (PROMs) with different features is available for people with inflammatory arthritis (IA).^1–5^ However, the needs and priorities of young people (aged 18–35 years) with IA regarding PROMs and their administration have never been systematically explored. Therefore, our project tackled the question whether PROMs commonly used in IA cover the perspectives and needs of young people. For this purpose, a task force (TF) guided by the 2014 EULAR Standardised Operating Procedures was convened.^6^ Given the limited literature on the perspectives of young people with IA regarding PROMs, a multinational focus group study^7^ and a subsequent online survey distributed across Europe^8^ replaced the conventional systematic literature review (online supplemental figure S1). The EULAR Council approved this research-based project approach. The TF was composed of 14 members from 8 European countries, with a strong representation of patient research partners. The TF met twice and, based on the results of the focus groups and survey, formulated four overarching principles (OPs), 8 points to consider (PtC) (table 1) and a research agenda (online supplemental table S1). Every TF member indicated agreement with a PtC or OP by formal voting (yes/no/abstain) during the second meeting and then anonymously scored their level of agreement (Numerical Rating Scale ranging from 0=‘no agreement’ to 10=‘absolute agreement’) after the meeting in a subsequent email round.^6^ OPs and PtC focused on the preferences of young patients with IA regarding the value of PROMs for shared decision making and monitoring, their content and mode of administration.

10.1136/rmdopen-2022-002576.supp1 Supplementary data

**Table 1:** Overarching principles and points to consider for including the perspective of young patients with IA into PROMs

	Overarching principles			LoA Mean (SD)
**A**	IA has a considerable impact on all aspects of the life of young people, and PROMs are useful to measure part of this impact			9.69 (±0.63), 100%≥8
**B**	The value of PROMs is optimised when young people with IA are informed and empowered			9.92 (0.28), 100%≥8
**C**	PROMs are useful when integrated in the communication between young people with IA and rheumatologists/other health professionals who are involved in their care.			9.77 (0.44), 100%≥8
**D**	PROMs inform shared decision-making for young people with IA.			9.31 (0.18), 92%≥8
	**Points to consider**	**LoE**	**Strength of recommendation**	**LoA** **Mean (SD**)
**1**	Young people with IA should be informed about the purpose and relevance of PROMs.	5	D	9.85 (0.37), 100%≥8
**2**	Young people with IA should have the possibility to access their personal PROM data.	5	D	9.31 (1.18), 85%≥8
**3**	Healthcare providers and young people with IA should discuss the results of PROMs and integrate them into shared decision making.	5	D	9.78 (0.44), 100%≥8
**4**	Different PROMs assess various domains and should therefore be used to cover a broad spectrum of the disease.	5	D	9.15 (1.82), 85%≥8
**5**	Healthcare providers should ascertain the willingness of young people with IA to talk about issues such as body image and life plans, and discuss these domains respecting the patients’ preferences.	5	D	9.54 (1.13), 92%≥8
**6**	The assessment of a young person with IA should encompass items and domains of his/her daily life such as psychosocial issues, participation in social activities, education/work, sports and using technologic devices.	5	C	9.46 (1.20), 92%≥8
**7**	The schedule of PROMs assessment should be agreed on by the healthcare provider and the young person with IA, to balance frequency versus inconvenience.	5	D	9.46 (1.05), 92%≥8
**8**	Online and e-solutions for PROMs should be used when possible and convenient for young people with IA.	5	D	9.92 (0.28), 100%≥8

Numbers in the column ‘LoA’ indicate the mean (SD) of the LoA, and the percentage of task force members with a LoA of at least 8 (0–10); based on the Oxford Centre for Evidence-Based Medicine classification

IA, inflammatory arthritis; LoA, level of agreement; LoE, level of evidence; PROMs, patient-reported outcome measures.

These EULAR PtC provide the first guidance to optimise the use of available PROMs for young people with IA. They should also serve as a companion for clinicians and researchers in rheumatology practice, and the relevant stakeholders when developing new PROMs and modifying existing PROMs. The OPs formulated by the TF underpin the value of PROMs as key elements to assess the impact of IA on the daily life of young people with IA, to aid the shared decision-making process.^9^ The PtC encompass several areas such as information/education on PROMs, their purpose and the use of their results (PtC 1–3, 7), daily life activities relevant to young people (PtC 4–6) and the use of technology for health-related purposes (PtC 8). Some PtC might be applicable to young patients and to patients aged above 35 years. However, despite these potential similarities, the PtC presented were developed from the needs and priorities identified by young people with IA regarding PROMs.^7^ Therefore, clinicians and researchers should strive to consider and embed the perspective of young people in the development of PROMS, to pave the way for a more inclusive, individualised and equal assessment of health, disease activity and well-being.

In summary, these are the first PtC for the use of PROMs in young patients with IA based on their own perspectives. We believe that the optimisation and harmonisation of PROMs used in daily practice could strengthen the relationship between patients and healthcare providers, facilitating shared decision making, and ultimately, the quality and experience of care for young people with IA.
