# Supplementary material for: EULAR points to consider for including the perspective of young patients with inflammatory arthritis into patient-reported outcomes measures
Source: RMD Open. 2022 Jul 29;8(2):e002576. doi: 10.1136/rmdopen-2022-002576 (PMC9345076; doi:10.1136/rmdopen-2022-002576)

Online Supplementary Table S1: Research agenda regarding patient-reported outcome measures for young people with inflammatory arthritis

- Identification of modifications to be applied to existing PROMs based on cross-checking qualitative and quantitative findings of commonly used PROs for young people with IA.
- Structured process of involving young people with IA as patient research partners in the development of future PROMs, and modification of existing PROMs.
- Validation of functional questionnaires focusing on those aged 35 years or younger, for further adaption to cover young peoples' issues, which have not been previously assessed.
- Agreement on which available PROMs should be used in clinical practice (as a core set) demonstrating value to young people with IA.

IA: inflammatory arthritis; PRO: patient-reported outcome; PROM: patient-reported outcome measure

Online Supplementary Figure S1. Timeline of the project to develop points to consider for including the perspective of young patients with inflammatory arthritis into patient-reported outcomes measures

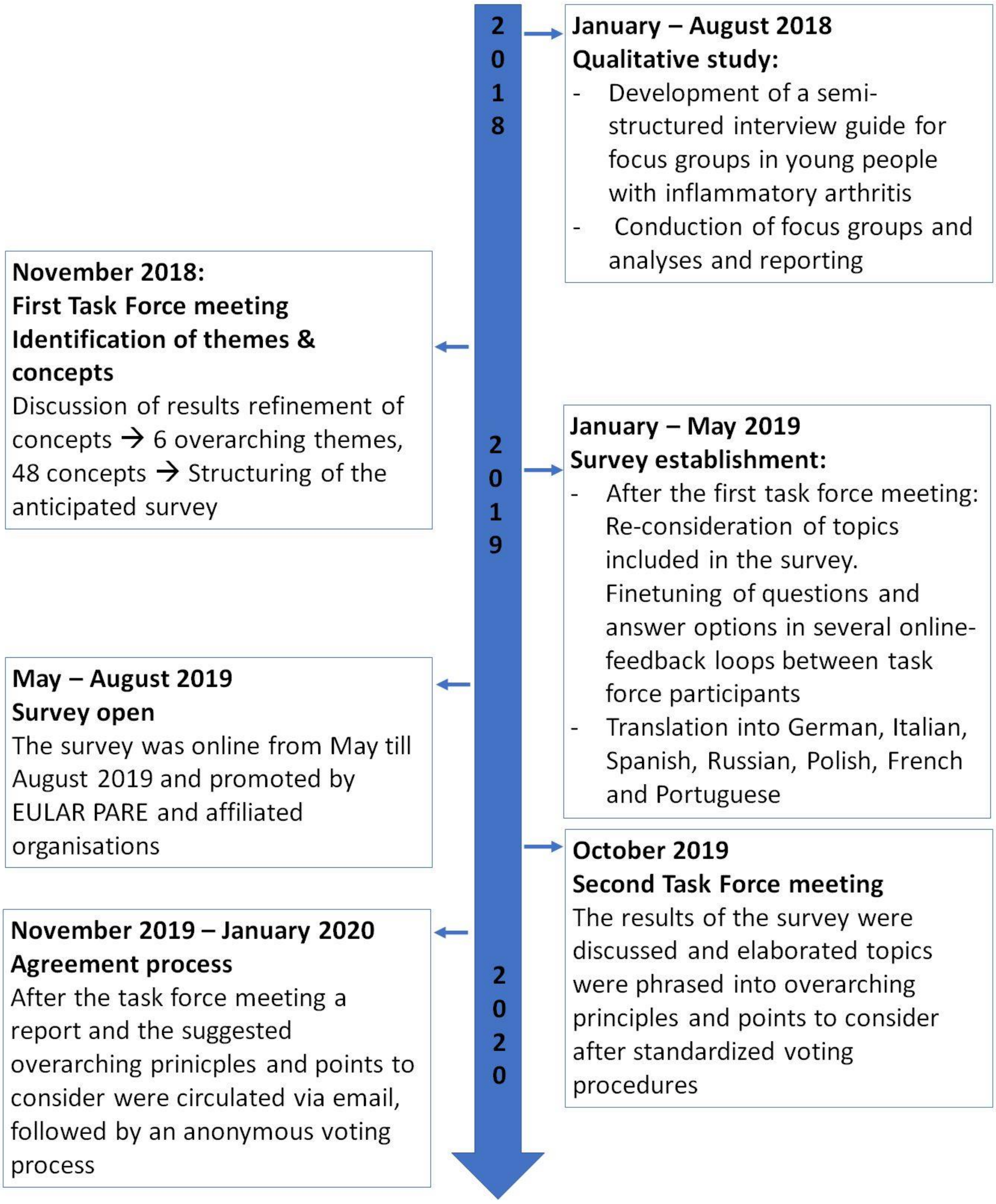

Supplement: Supplementary data [file rmdopen-2022-002576supp001.pdf]
